# Curcumin-Mediated Apoptotic Cell Death in Papillary Thyroid Cancer and Cancer Stem-Like Cells through Targeting of the JAK/STAT3 Signaling Pathway

**DOI:** 10.3390/ijms21020438

**Published:** 2020-01-09

**Authors:** Abdul Q. Khan, Eiman I. Ahmed, Noor Elareer, Hamna Fathima, Kirti S. Prabhu, Kodappully S. Siveen, Michal Kulinski, Fouad Azizi, Said Dermime, Aamir Ahmad, Martin Steinhoff, Shahab Uddin

**Affiliations:** 1 Translational Research Institute, Academic Health System, Hamad Medical Corporation, Doha 3050, Qatar; AKhan42@hamad.qa (A.Q.K.); emoibrahim04@gmail.com (E.I.A.); NElareer@hamad.qa (N.E.); Hamnafathima2001@gmail.com (H.F.); KPrabhu@hamad.qa (K.S.P.); SSivaraman@hamad.qa (K.S.S.); MKulinski@hamad.qa (M.K.); FAzizi@hamad.qa (F.A.); MSteinhoff@hamad.qa (M.S.); 2 National Centre for Cancer Care and Research, Hamad Medical Corporation, Doha 3050, Qatar; sdermime@hamad.qa; 3 Department of Medicine, University of Alabama at Birmingham, Birmingham, AL 35205, USA; 4 Department of Dermatology and Venereology, Hamad Medical Corporation, Doha 3050, Qatar; 5 Department of Medicine, Weill Cornell Medicine-Qatar, Qatar Foundation-Education City, Doha 24144, Qatar; 6 Department of Medicine, Weill Cornell Medicine, 1300 York Avenue, New York, NY 10065, USA; 7 College of Medicine, Qatar University, Doha 2713, Qatar

**Keywords:** thyroid cancer, curcumin, cisplatin, STAT3, cell proliferation, cancer stem cells, apoptosis

## Abstract

The constitutive activation of Janus Kinase/Signal Transducer and Activator of Transcription (JAK/STAT) signal transduction is well elucidated in STAT3-mediated oncogenesis related to thyroid cancer and is considered to be a plausible therapeutic target. Hence, we investigated whether curcumin, a natural compound, can target the JAK/STAT3 signaling pathway to induce cytotoxic effects in papillary thyroid cancer (PTC) cell lines (BCPAP and TPC-1) and derived thyroid cancer stem-like cells (thyrospheres). Curcumin suppressed PTC cell survival in a dose-dependent manner via the induction of caspase-mediated apoptosis and caused the attenuation of constitutively active STAT3 (the dephosphorylation of Tyr705–STAT3) without affecting STAT3. Gene silencing with STAT3-specific siRNA showed the modulation of genes associated with cell growth and proliferation. The cotreatment of PTC cell lines with curcumin and cisplatin synergistically potentiated cytotoxic effects via the suppression of JAK/STAT3 activity along with the inhibition of antiapoptotic genes and the induction of proapoptotic genes, and it also suppressed the migration of PTC cells by downregulating matrix metalloproteinases and the inhibition of colony formation. Finally, thyrospheres treated with curcumin and cisplatin showed suppressed STAT3 phosphorylation, a reduced formation of thyrospheres, and the downregulated expression of stemness markers, in addition to apoptosis. The current study’s findings suggest that curcumin synergistically enhances the anticancer activity of cisplatin in PTC cells as well as in cancer stem-like cells by targeting STAT3, which suggests that curcumin combined with chemotherapeutic agents may provide better therapeutic outcomes.

## 1. Introduction

Malignancies of the human thyroid gland or thyroid carcinoma (TC) represent the most prevalent cancers of the endocrine system and has been ranked as the seventh common cancer type with relatively higher incidence rate [1]. A recent survey revealed that an increase in TC has been observed in the last few decades, which has put pressure on discovering effective management routes [2]. Although the treatment options available for thyroid malignancies are effective if diagnosed at an early stage (mainly surgery and radioiodine remnant ablation), there are more challenges in treating metastasized and relapsed forms of thyroid cancer, requiring a deep and thorough reinvestigation aimed at better therapeutic outcomes.

Deregulated functioning of cell survival signaling pathways is the main driver for the various hallmarks of carcinogenesis, including incessant cell proliferation and drug resistance. A number of oncogenic signaling molecules and pathways have been deregulated in all three types of thyroid cancer development. The role of pathways such as MAPK (mitogen-activated protein kinase), phospoinositide 3-kinase (PI3K/AKT), Wingless/ β-catenin (WNT/β-catenin), Nuclear Factor kappa-light-chain-enhancer of activated B cells (NF-кB), proto-oncogene B-Raf (B-Raf), Proto-Oncogene Proteins p21(Ras), Phosphatase and tensin homolog (PTEN) have been well elucidated [1,3,4]. Various investigations have shown that PI3K and MAPK are the most centric cascades of thyroid carcinogenesis, reflecting a web of complex and interconnected molecules and pathways. 

One of the pathways is the Janus kinase (JAK)/signal transducer and activator of transcription (STAT) pathway, which has been studied less often in terms of thyroid malignancies. JAK/STAT3 signal transduction is vital for normal cellular homeostasis, as it regulates cell proliferation, growth, apoptosis, differentiation, and other important cellular biological phenomenons. In most of the human malignancies, aberrantly activated JAK-induced constitutive upregulation of STAT transcription factors has been shown to play critical role in cancer development and associated challenges [5]. Further, a number of reports have also revealed that the cytokine-induced activation of STAT3 is associated with the evasion of apoptosis, among other cancer hallmarks [6,7,8]. There is a strong correlation between STAT3 overexpression and the pathogenesis of thyroid cancer, as the dysregulated activation of STAT3 has been reported in human specimens of thyroid cancers [9,10].

Cancer stem cells (CSCs) are a small population of tumor cells that are critically implicated in cancer pathogenesis and in various drawbacks to cancer therapy, such as disease recurrence and resistance. Earlier reports revealed that CSCs are the primary cause of cancer resistance towards anticancer drugs treatment including cisplatin-based chemotherapy [11,12]. Furthermore, thyroid CSCs, which are present in very minute quantities, are known to play a critical role in the recurrence of tumors; tumor initiation, growth, and metastasis; and resistance to therapy [13,14]: hence, the targeting of these cells is important for better therapeutic outcomes.

Curcumin (diferuloylmethane) is one of the most studied phytochemicals found in plants *Curcuma longa* (Linn) and has been shown to possess strong antioxidant, anti-inflammatory, and anticancer potential over a range of human cancers [15,16]. In various cancers, curcumin has been shown to inhibit growth and proliferation of cancer cells by targeting various survival pathways, including JAK/STAT3, PI3-kinase/AKT, Transforming growth factor beta (TGF-β), Epidermal Growth Factor Receptor (EFGR), and NF-кB [17,18,19,20,21]. In addition, there is attenuation of the transcriptional expression of regulatory proteins associated with programmed cell death or apoptosis. Further, it is also involved in the modulation of aberrant epigenetics mechanisms and the expression of noncoding RNA [20]. Interestingly, a number of studies have shown that curcumin exerts its pharmacological action by targeting JAK/STAT3 signaling [22,23,24]. Anticancer drugs, such as cisplatin, that are used in chemotherapy (one of the therapeutic options used by clinicians) have been found to be associated with various critical complications, including drug resistance in papillary thyroid cancer (PTC) patients [11], and the available literature has shown that a number of natural products, including curcumin, have shown synergistic action with anticancer drugs [25,26,27,28].

Interleukins are central secretory molecules that are well known for their integral role in biological homeostasis (including thyroid functioning and hormone release) and that are regulated by tight regulatory mechanisms [29]. IL6, an important cytokine, has been shown to mediate diverse biological functions including normal cellular growth and immune response through activation of STAT3 while its aberrant secretion is known to associated with the pathogenesis of various human diseases including thyroid cancer [30,31].

In the current study, we elucidated for the first time the antiproliferative action of curcumin alone and in combination with cisplatin in human thyroid cancer cell lines by targeting survival pathways. Cisplatin alone has been found to be associated with drawbacks in PTC patients, so we wanted to see whether the cotreatment of curcumin with cisplatin in PTC cells (BCPAP and TPC-1) enhanced the anticancer potential of cisplatin, which would be of great importance for the development of drugs with safe and effective doses. Further, we also studied the effect of curcumin and cisplatin on the stemness of cancer stem cells. In addition, we also explored the role of IL6 in the stimulation of STAT3 and in the growth and proliferation of PTC cancer cells. Our data showed that curcumin synergistically potentiated the chemotherapeutic potential of cisplatin, as it enhanced reduction in cell viability, proliferation, and apoptosis through the downregulation of JAK/STAT3-mediated cancer stemness.

## 2. Results

### 2.1. Curcumin-Mediated Inhibition of Cell Proliferation and Apoptosis in PTC Cells

**Figure 1:**
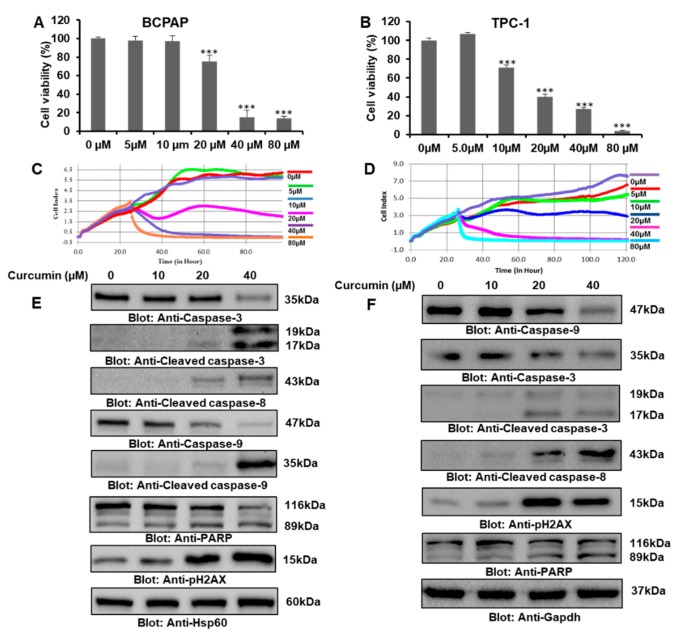
Curcumin-mediated inhibition of cell proliferation and apoptosis in PTC cells. (**A**,**B**) Curcumin inhibited the cell viability of PTC cell lines BCPAP and TPC-1. PTC cells were treated with 0 µM, 5 µM, 10 µM, 20 µM, 40 µM, and 80 µM of curcumin for 24 h. A cell proliferation assay was performed using a Cell Counting Kit-8 (CCK-8), as described in “Materials and Methods”. Values are expressed as the mean +/− the SD (standard deviation) of at least six replicates, with a *p*-value of *** *p* <0.001. (**C**,**D**) Real-time cell proliferation (cell index) analysis of BCPAP and TPC-1 cells, respectively, treated with different doses of curcumin. Cells were cultured on electrodes and treated with curcumin followed by the monitoring of real-time cell growth, as mentioned in the material and methods section. (**E**,**F**) Curcumin mediated the inhibition of BCPAP and TPC-1 cell growth and proliferation through apoptosis. PTC cell lines were treated with different doses of curcumin for 24 h, followed by cell lysis and immunoblotting. Equal amount of proteins were separated using SDS-PAGE, transferred to a Polyvinylidene fluoride or polyvinylidene difluoride (PVDF) membrane, and immunoblotted with antibodies against caspase-3, cleaved caspase-3, cleaved caspase-8, caspase-9, cleaved caspase-9, poly (ADP-ribose) polymerase (PARP), phosphor-histone family member X (p-H2AX), heat shock protein 60 (Hsp60), and Gapdh.

Initially, we investigated the effect of curcumin alone on the cell viability of thyroid cancer cells. BCPAP and TPC-1 cells were treated with gradient doses of curcumin for 24 h, and the cell viability of treated and untreated cell lines was assayed using Cell Counting Kit-8 (CCK-8). Our data analysis revealed that curcumin inhibited BCPAP and TPC-1 cell viability in a dose-dependent manner (Figure 1A,B, respectively). Curcumin at doses of 20 μM and above resulted in a significant inhibition of BCPAP cell viability, while in the case of TPC-1, concentrations of 10 μM and above led to a significant reduction in cell viability. Further, the effect of curcumin on cell proliferation in real time through xCELLigence real-time cell analysis (RTCA) showed that curcumin treatment suppressed the growth index of thyroid cell lines (Figure 1B,C). Then, we wanted to know whether curcumin-mediated cell cycle arrest would lead to apoptosis, so in a series of experiments, we first checked the effect of curcumin on the cell cycle, and our data demonstrated a remarkable increase in the SubG0/G1 phase of BCPAP cell lines treated with curcumin for 24 h (Supplementary Materials, Figure S1A). We also showed the temporal effect of curcumin on various stages of the cell cycle at 48 and 72 h (Supplementary Materials, Figure S1A). Annexin V/PI dual staining further supported the induction of apoptosis, as cells treated with 10 μM, 20 μM, and 40 μM of curcumin for 24 h showed an increased percentage of apoptosis compared to control cells (Supplementary Materials, Figure S1B). Then, we further investigated the underlying molecular mechanisms, and a series of experiments was done to assess the level of various signaling proteins associated with apoptosis in response to curcumin in PTC cell lines. PTC cells were treated with 10 μM, 20 μM, and 40 μM of curcumin for 24 h, and an expression analysis of various apoptotic markers was done, which clearly showed that curcumin treatment caused apoptosis via the activation of caspases through the mitochondrial apoptotic pathway, as treatment with curcumin led to a significant decrease in the mitochondrial membrane potential (MMP) of BCPAP, as shown in the Supplementary Materials, Figure S1C,D. Western blot data showed that curcumin upregulated the activation or expression of caspase-3, cleaved caspase-3, cleaved caspase-8, capase-9, cleaved caspase-9, and PARP and the phosphorylation of H2AX (Figure 1E,F), which suggests that curcumin mediates the inhibition of PTC cell growth and proliferation via the induction of apoptosis.

### 2.2. Curcumin Suppressed the Constitutive JAK/STAT3 Signaling Pathway and Upregulated the ROS Level to Induce Apoptosis

**Figure 2:**
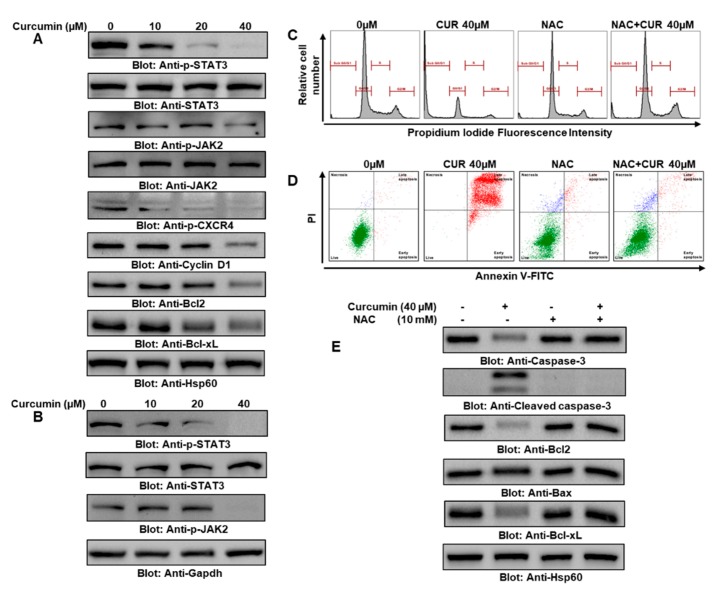
Curcumin suppressed the constitutive JAK/STAT3 signaling pathway and upregulated reactive oxygen species (ROS) content to induce apoptosis. (**A**) BCPAP and (**B**) TPC-1 cells were treated with 10 µM, 20 µM, and 40 µM of curcumin for 24 h. After cell lysis, equal amounts of proteins were separated by SDS-PAGE, transferred to a PVDF membrane, and immunoblotted with antibodies against p-STAT3, STAT3, p-JAK2, JAK2, Cyclin D, pCXCR4, Bcl2, Bclxl, and Hsp60. (**C**) Curcumin induced ROS generation and oxidative stress, which are involved in cell cycle arrest and apoptotic cell death in PTC cells. *N*-acetyl cysteine (NAC) pretreatment with abrogated curcumin induced an increase in the SubG0 fraction in PTC cells. BCPAP cells were pretreated with 10 mM NAC followed by 40 µM curcumin for 24 h, and the cell cycle fraction was measured by flow cytometry. (**D**) BCPAP cells were pretreated with 10 mM NAC followed by 40 µM curcumin for 24 h, and apoptosis was measured by staining with fluorescein-conjugated Annexin V and propidium iodide (PI) and analyzed by flow cytometry. Data are expressed as the mean ± the SD. (**E**) NAC pretreatment in PTC cells reversed curcumin-mediated apoptosis. BCPAP cells were pretreated with 10 mM NAC and then subsequently treated with 40 µM curcumin. Cells were lysed and separated using SDS-PAGE, transferred to a PVDF membrane, and immunoblotted with antibodies such as caspase-3, cleaved caspase-3, Bcl2, Bax, and Hsp60. Curcumin, CUR; cisplatin, CIS.

We next wanted to find out about the constitutive phosphorylation of STAT3 in PTC cells. BCPAP and TPC-1 cells were challenged with 10 μM, 20 μM, and 40 μM of curcumin for 24 h. The phosphorylation status of try705 residue was determined by immunoblotting using anti-p-STAT3–try705 antibody. As shown in Figure 2A,B, constitutively phosphorylated STAT3 was seen in untreated BCPAP and TPC-1 cells, and curcumin treatment resulted in the significant dephosphorylation of STAT3 (Supplementary Materials, Figure S2A,B) in a concentration-dependent manner. Interestingly, curcumin did not show any effect on the expression of the STAT3 protein in the BCPAP and TPC-1 cell lines, as shown in Figure 2A,B respectively. The activation of STAT3 is regulated by nonreceptor tyrosine kinases (JAKs), and we investigated if JAK2 is involved in the curcumin-mediated dephosphorylation of STAT3 in PTC cells (as shown in Figure 2A,B): the presence of constitutively active JAK2 in BCPAP and TPC-1 cells and the curcumin-mediated inhibition of JAK2 phosphorylation suggested the involvement of JAK2/STAT3 signaling in curcumin-mediated apoptosis (as represented in the Supplementary Materials, Figure 2C (BCPAP) and 2D (TPC-1). Further, curcumin treatment in BCPAP cells downregulated the expression of C-X-C chemokine receptor type 4 (CXCR4) (Figure 2A). A number of gene products are regulated by the constitutive activation of JAK2/STAT3, which is involved in many cellular functions, including cell proliferation and apoptosis. Therefore, we determined whether the curcumin-mediated inactivation of JAK2/STAT3 could influence the expression of these genes in both types of human PTC cell lines. As shown in Figure 2A, the curcumin treatment of BCPAP cells suppressed the expression of regulatory proteins associated with growth and cell proliferation in a dose-dependent manner. 

Next, we wanted to explore the role of ROS or oxidative stress in the curcumin-induced apoptotic cell death of PTC cell lines, because ROS play a vital role in cancer therapy. To determine whether ROS are involved in the curcumin-mediated apoptotic death of PTC cells, BCPAP and TPC-1 cells were pretreated with 10 mM of *N*-acetyl cysteine (NAC), a universal ROS scavenger, and subsequently cells were treated with 40 μM of curcumin for 24 h. A significant increase in the SubG0/G1 fraction of cell cycle and apoptosis was observed in the curcumin-treated BCPAP cells, while pretreatment with NAC effectively suppressed cell cycle arrest and apoptosis (Figure 2C,D). Further expression analyses of different proteins using western blot analysis and densitometry data showed that the activation of caspase-3 and cleaved caspase-3, the downregulation of antiapoptotic molecule Bcl2 and Bcl-xL, and the upregulation of proapoptotic marker Bax were markedly reversed by NAC, suggesting that ROS are vital in the curcumin-induced apoptosis of BCPAP (Figure 2E, Supplementary Materials, Figure S2E,G) and TPC-1 cells (Supplementary Materials, Figure S2H). Densitometry data showed a markedly increased Bax/Bcl2 ratio in the BCPAP cells treated with curcumin compared to the control, while the NAC and NAC + curcumin groups showed a suppressed Bax/Bcl2 ratio compared to curcumin-treated cells, suggesting that ROS are major players in curcumin-mediated apoptosis in PTC cells (Supplementary Materials, Figure S2E).

### 2.3. Synergistic Activity of Curcumin and Cisplatin in PTC Cell Lines

**Figure 3:**
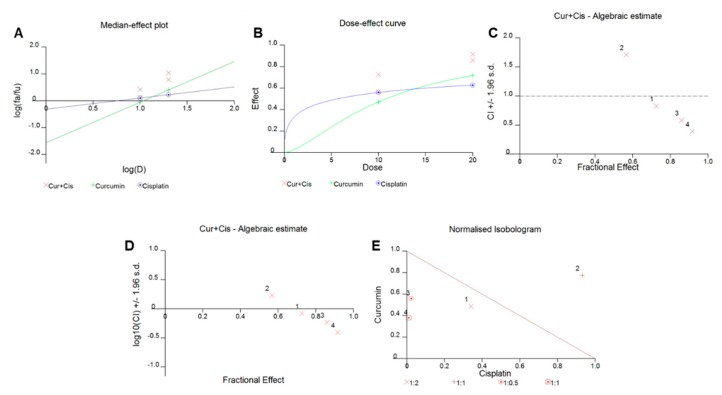
Curcumin synergistically enhanced the antiproliferative response of cisplatin in PTC cells. BCPAP cells were treated with various combinations of curcumin and cisplatin for 24 h, and graphs were generated using Calcusyn software, as described by Chou and Talalay. Briefly, curcumin and cisplatin were combined, and at least four different combinations were used to treat PTC cells for 24 h. A cell proliferation assay was performed using a CCK8 kit, as described in the materials and methods section, and data are expressed as the mean +/− the SD of at least six replicates. Different types of graphs, using Calcusyn software (as described by Chou and Talalay), including (**A**) a median dose effect graph, where the *y* axis represents the scale of *fa* (the fraction affected by the dose) and *fu* (the fraction unaffected by dose); (**B**) a graph representing the dose effect curve of curcumin and cisplatin alone and in combination; (**C**,**D**) graphs representing the combination index (CI) value of the curcumin and cisplatin combination; and (**E**) a graph representing an isobologram of the curcumin and cisplatin interaction, were generated. CUR: curcumin; CIS: cisplatin.

We sought to determine whether the cotreatment of BCPAP and TPC-1 cells with curcumin and cisplatin could potentiate anticancer effects in PTC cells. We performed a series of experiments to find out optimal concentrations that could potentiate the inhibition of cell viability, motility, and colony formation and trigger apoptotic cell death in PTC cells as well as in PTC cancer stem cells. To determine the various combination indices of curcumin and cisplatin in PTC cells, we generated various graphs of curcumin with cisplatin in PTC cells (Figure 3A–E) by using Calcusyn software based on *Chou* and *Talalay’s* methodology [32]. In brief, the different outcomes of the curcumin and cisplatin interaction were determined using the median-effect principle of *Chou* and *Talalay’s* mathematic model, which is based on the median-effect principle formula *fa/fu = [D/Dm]m*, where *D* equals the dose of the drug, *fa* is the fraction affected by the dose, *fu* is the unaffected fraction, *Dm* is the drug concentration required for 50% inhibition (IC50), and *m* is the slope of the dose effect. The combination index (CI) represents the quantitative interaction between drugs and CI values < 1 (synergy), = 1 (additive effect), and > 1 (antagonism), as explained by Chou and Talalay. Interestingly, we observed that the CI values of three out of four of the different combinations of curcumin and cisplatin were less than one. Our data also showed that the combination of curcumin and cisplatin showed a significant inhibitory effect on the viability of PTC cells compared to the individual drug effect. 

### 2.4. Cotreatment with Curcumin and Cisplatin Augmented the Inhibition of Cell Viability

**Figure 4:**
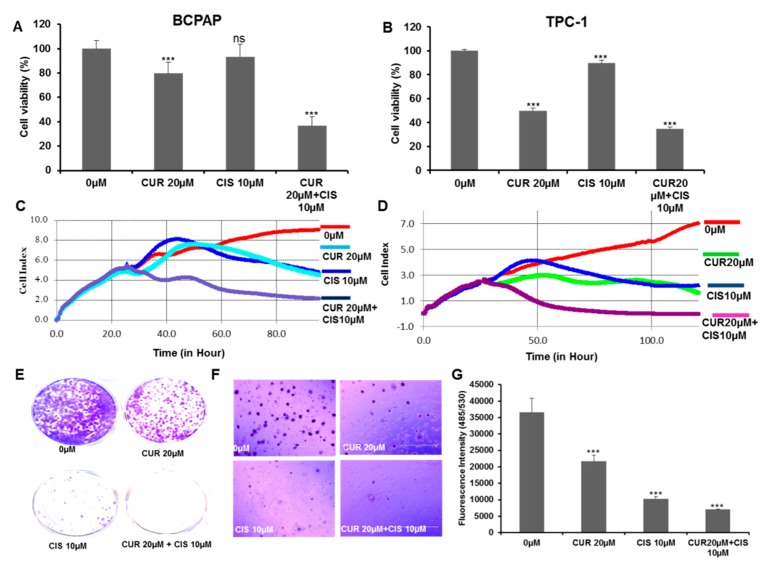
Cotreatment of curcumin with cisplatin augmented the inhibition of cell growth and proliferation and suppressed the motility of PTC cells. Curcumin synergistically enhanced the growth inhibitory potential of cisplatin in BCPAP (**A**) and TPC-1 (**B**) cells. BCPAP and TPC-1 cells were treated with 0 µM or 20 µM of curcumin and 10 µM cisplatin alone and in combination for 24 h. A cell proliferation assay was performed using a CCK8 kit, as described in the materials and methods section. Values were expressed as the mean +/− the SD of at least six replicates, with a *p*-value od *** *p* < 0.001. (**C**,**D**) Real-time cell proliferation (cell index) analysis of BCPAP and TPC-1 cells treated with curcumin and cisplatin, respectively. BCPAP and TPC-1 cells were cultured on electrodes and treated with 20 µM curcumin and 10 µM cisplatin alone and in combination followed by the monitoring of real-time cell growth, as mentioned in the material and methods section. (**E**–**G**) Curcumin synergistically inhibited the colony formation potential of cisplatin. BCPAP cells were cultured in a six-well plate with and without agar and treated with 0 µM or 20 µM curcumin and 10 µM of cisplatin alone and in combination for one to two weeks. Colonies were stained with crystal violet, and fluorescence intensity using CyQuant dye was measured at 485/530 nm. Data are presented as the mean +/− the SD, *** *p* < 0.001. Curcumin (CUR), cisplatin (CIS).

Using the Chou and Talalay method, we optimized the doses of curcumin and cisplatin for maximal synergistic effects on human PTC cells. Further, we did a series of experiments to assess the comparative effects of curcumin and the chemotherapeutic drug cisplatin alone as well as in combination on BCPAP and TPC-1 cells. The viability of PTC cells was determined by CCK8 assays, as described in the methods section. The treatment of PTC cells with curcumin and cisplatin markedly inhibited the proliferation of BCPAP and TPC-1 compared to curcumin or cisplatin alone, as shown in Figure 4A,B respectively. In addition, we also performed a real-time cell proliferation analysis after curcumin and cisplatin treatment by using a real-time cell analyzer (RTCA) and found that the combination of curcumin and cisplatin affected the cell index to a greater extent than either alone in BCPAP and TPC-1 cells, as shown in Figure 4C,D respectively. Overall, the CCK-8 and RTCA-based findings on cell proliferation and growth suggest that curcumin markedly sensitized PTC cells toward cisplatin.

Further, our data also revealed that curcumin synergistically inhibited the colony formation potential of PTC cells. BCPAP cells that were treated with 0 µM or 20 µM of curcumin and 10 µM of cisplatin alone and in combination and were grown for about two weeks developed significantly fewer numbers of colonies compared to the control (Figure 4E–G). In addition, we also wanted to explore the effect of curcumin and cisplatin cotreatment on PTC cell migration by targeting CXCR4 and matrix metalloproteinases (MMPs), which are often associated with proliferation, metastasis, and angiogenesis. BCPAP and TPC-1 cells were treated with 0 µM or 20 µM curcumin and 10 µM of cisplatin alone and in combination for 24 h. Cell migration was assayed by a wound-healing assay, and we found that treatment with both curcumin and cisplatin markedly increased the inhibition of the migration of BCPAP and TPC-1 cells compared to drug treatment alone, as shown in the Supplementary Materials, Figure S3A,B, respectively. We also investigated whether the cotreatment of BCPAP cells with 0 µM or 20 µM of curcumin and 10 µM of cisplatin alone and in combination suppressed the expression of p-CXCR4, MMP9, and MMP2, which are critical to the invasion and migration ability of cells. As shown in the Supplementary Materials, Figure S3C–E, the combination treatment of PTC cells with curcumin and cisplatin downregulated the expression of p-CXCR4, MMP9, and MMP2. 

### 2.5. Cotreatment of Curcumin and Cisplatin Enhanced Apoptosis via the Downregulation of the JAK/STAT Survival Pathway in PTC Cells

**Figure 5:**
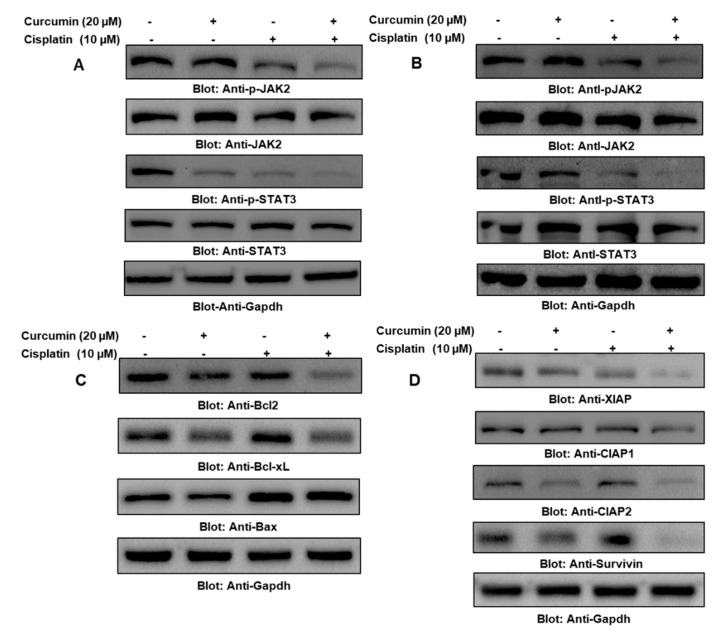
Curcumin and cisplatin synergistically enhanced the inhibition of JAK/STAT3 and Inbibitor of apoptosis proteins (IAPs) and also modulated apoptotic regulators. (**A**,**B**) The cotreatment of curcumin and cisplatin enhanced the downregulation of JAK/STAT3 in BCPAP and TPC-1 cells. PTC cell lines were treated with 0 µM or 20 µM of curcumin and 10 µM of cisplatin alone and in combination, and after cell lysis, equal amounts of proteins were separated by SDS-PAGE, transferred to a PVDF membrane, and immunoblotted with antibodies of p-STAT3, STAT3, p-JAK2, JAK2, and Gapdh (**C**) Curcumin and cisplatin modulated the expression of antiapoptotic and proapoptotic proteins in PTC cells. BCPAP cells were treated with 0 µM and 20 µM of curcumin and 10 µM of cisplatin alone and in combination, and after cell lysis, equal amounts of proteins were separated by SDS-PAGE, transferred to a PVDF membrane, and immunoblotted with antibodies of Bcl2, Bclxl, Bax, and Gapdh. (**D**) Curcumin and cisplatin synergistically downregulated IAP expression in PTC cells. BCPAP cells were treated with 0 µM or 20 µM of curcumin and 10 µM of cisplatin alone and in combination, and after cell lysis, equal amounts of proteins were separated by SDS-PAGE, transferred to a PVDF membrane, and immunoblotted with antibodies of XIAP, CIAP1, CIAP2, Survivin, and Gapdh.

Constitutively activated STAT3 plays a vital role in oncogenic manifestations in most human cancers, including thyroid cancer. Hence, we treated BCPAP and TPC-1 cells with curcumin and cisplatin alone and in combination for 24 h, as shown in Figure 5A,B, respectively. Immunoblotting data analysis revealed that there was a significant downregulation in the expression of p-STAT3 in all three experimental conditions and that it was most effective in cells treated with both curcumin and cisplatin in the BCPAP and TPC-1 cell lines, as shown in the Supplementary Materials, Figure S4A,B, respectively. Further, to support our data, we also immunoblotted total STAT3 and found that the treatment conditions did not affect the total STAT3 status. As the cellular status of STAT3 is regulated by the nonreceptor tyrosine kinase JAK2, we checked and analyzed the JAK2 protein in BCPAP and TPC-1 treated with 0 µM and 20 µM of curcumin and 10 µM of cisplatin alone and in combination for 24 h and found a marked dephosphorylation of JAK2, as represented in Figure 5A (BCPAP) and Figure 5B (TPC-1) and in the Supplementary Materials, Figure S4C (BCPAP) and 4D (TPC-1). Additionally, we also checked the effect of the curcumin- and cisplatin-mediated downregulation of STAT3 activation on its downstream targets, including Bcl2, Bcl-xL, and XIAP, which are well-known regulators of cell growth and proliferation. Our results depicted that treatment of BCPAP cells with 0 µM and 20 µM of curcumin and 10 µM of cisplatin alone and in combination downregulated antiapoptotic proteins and inhibitors of apoptosis proteins, while there was an upregulated expression of proapoptotic proteins (Figure 5C). cIAPs are the family of proteins that is involved in the regulation of caspase-mediated apoptosis, so we determined the effect of curcumin and cisplatin alone and in combination on the members of the IAP family. The PTC cell line BCPAP was treated with curcumin and cisplatin alone and in combination for 24 h, and the expression of XIAP, cIAP1, and cIAP2 was determined through immunoblotting. As shown in Figure 5D, a combination of curcumin and cisplatin treatment induced the downregulation of XIAP compared to cells treated with curcumin or cisplatin alone, and the densitometric data showed that there was a significant change in cIAP-2 expression, while there was no significant change in the expression of cIAP-1 (Supplementary Materials, Figure S4E,F). Overall, these findings suggest that curcumin- and cisplatin-mediated apoptosis involves XIAP, cIAPs, and survivin in PTC cells, most likely through the activation of mitochondrial apoptotic signaling. 

Members of the Bcl-2 family are the key regulators of cell proliferation and apoptosis, as they maintain the homeostatic balance between proapoptotic and antiapoptotic molecules. Imbalances or disturbances in these proteins levels lead to the stimulation or prevention of cell death. We aimed to determine whether the treatment of PTC cells with curcumin and cisplatin enhanced the expression levels of Bax and suppressed the expression of Bcl-2. As shown in Figure 5C, it was observed that the treatment of BCPAP cells with curcumin and cisplatin caused a decrease in the expression levels of antiapoptotic Bcl-2 protein, with a subsequent increase in the expression level of the proapoptotic protein Bax, indicating that the curcumin- and cisplatin-mediated over expression of Bax and the downregulation of Bcl2 played a role in apoptotic cellular death. Low Bax and high Bcl-2 expression have been shown to cause resistance, whereas a high level of Bax and low Bcl-2 expression are known to enhance sensitivity to drug-induced apoptosis. Our data showed that curcumin and cisplatin treatment caused an increased Bax/Bcl2 ratio, suggesting that the curcumin-mediated overexpression of Bax and the downregulation of Bcl2 play a role in curcumin- and cisplatin-induced apoptosis.

**Figure 6:**
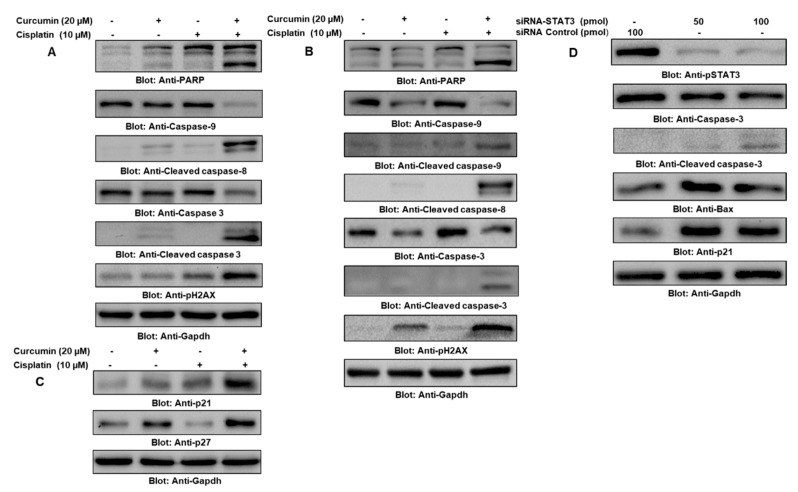
Cotreatment of curcumin with cisplatin-enhanced apoptosis of PTC cells via STAT3 downregulation. Curcumin potentiated cisplatin-induced growth arrest and inhibited the proliferation of BCPAP (**A**) and TPC-1 (**B**) cells through apoptosis. PTC cells were cultured and treated with 0 µM or 20 µM of curcumin and 10 µM of cisplatin alone and in combination for 24 h. Cells were lysed and separated using SDS-PAGE, transferred to a PVDF membrane, and immunoblotted with antibodies such as caspase-9, cleaved caspase-9, cleaved caspase-8, caspase-3, cleaved caspase-3, PARP, p-H2AX, and Gapdh. (**C**) Curcumin synergistically potentiated cisplatin-induced growth inhibition by stimulating the expression of cyclin-dependent kinase inhibitor proteins (CDKIs) in PTC cells. BCPAP cells were treated with 0 µM and 20 µM of curcumin and 10 µM of cisplatin alone and in combination for 24 h, followed by cell lysis and immunoblotting. Equal amounts of proteins were separated using SDS-PAGE, transferred to a PVDF membrane, and immunoblotted with antibodies against p27, p21, and Gapdh. (**D**) The effect of siRNA-mediated knockdown of STAT3 on p-STAT3, CDKIs, and caspases. BCPAP cells were transfected with either control (100 pmol) or STAT3-specific siRNA (50 or 100 pmol). Cell extracts were separated using SDS-PAGE, transferred to a PVDF membrane, and immunoblotted with antibodies against p-STAT3, caspase-3, cleaved caspase-3, Bax, p21, and Gapdh.

Programmed cell death, or apoptosis, is a complex heterogeneous vital mechanism that is involved in the maintenance of cellular and physiological functioning. In the present study, we demonstrated the underlying mechanism that is activated in curcumin- and cisplatin-induced apoptosis in PTC cell lines. To do so, first, we sought to determine the role of mitochondria-mediated apoptosis in curcumin- and cisplatin-treated BCPAP cells by measuring the mitochondrial membrane potential (MMP) using JCI dye through flow cytometry. Our data revealed that treatment with curcumin and cisplatin alone and in combination caused enhanced JCI staining that depicted the loss of MMP. Interestingly, the loss of MMP was more significant in cells treated with curcumin and cisplatin with respect to treatment with either alone, suggesting that curcumin sensitizes cells to cisplatin (Supplementary Materials, Figure S5A). Further, our results also showed that BCPAP and TPC-1 cells treated with curcumin and cisplatin alone and in combination caused apoptosis, as shown in Figure 6A,B respectively. We also found that the treatment of BCPAP cells with curcumin and cisplatin in combination markedly modulated the expression of cyclin-dependent kinase inhibitor proteins (CDKIs) compared to individual treatment, which was further supported by the densitometry data analysis (Figure 6C) and (Supplementary Materials, Figure S5B,C). Further, to demonstrate the involvement of STAT3 in the curcumin- and cisplatin-induced inhibition of cell growth and proliferation, we genetically knocked down STAT3 and checked the expression of proteins associated with the regulation of cell growth and proliferation, and our analysis of the results supported the role of STAT3 in the growth of PTC cells (Figure 6D).

### 2.6. ROS Were Central in Curcumin- and Cisplatin Cotreatment-Induced Apoptosis in PTC Cells

**Figure 7:**
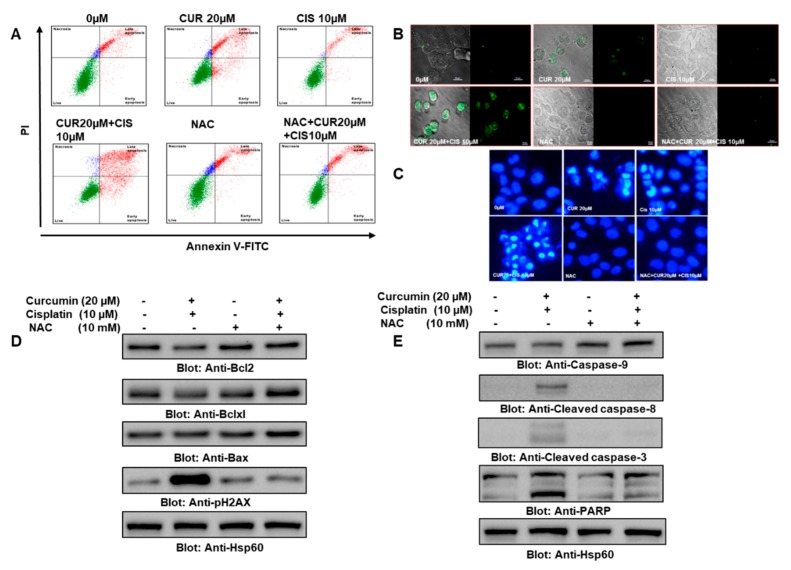
Curcumin potentiated the cisplatin-mediated apoptosis of PTC cells via enhanced oxidative stress. (**A**) NAC pretreatment prevented the curcumin- and cisplatin-mediated apoptosis of PTC cells. BCPAP cells were treated with 0 µM and 20 µM of curcumin and 10 µM cisplatin, 20 µM curcumin plus 10 µM cisplatin, NAC (10 mM) and NAC plus 20 µM curcumin and 10 µM cisplatin for 24 h; cells were subsequently stained with fluorescein-conjugated Annexin-V and propidium iodide (PI) and analyzed by flow cytometry; apoptosis was measured; and the percentage of apoptosis relative to untreated cells was calculated. (**B**) NAC pretreatment prevented curcumin- and cisplatin-induced increased ROS generation in PTC cells. BCPAP cells were treated with 0 µM or 20 µM of curcumin and 10 µM of cisplatin alone and in combination or were treated with NAC (10 mM) and NAC + curcumin and cisplatin for 24 h; cells were fixed and subsequently stained with 2′,7′ –dichlorofluorescin diacetate (DCFDA); and images were taken through confocal microscopy at 60X magnification (scale bar: 25 µm). DCFDA is well known for measuring the various ROS species: once it diffuses inside the cells, it gets deacetylated by cellular esterases, leading to the formation of a non-fluorescent compound. Next, cellular ROS oxidized DCFDA into a highly fluorescent compound known as dichlorofluorescein (DCF), which can be detected by fluorescence spectroscopy. (**C**) NAC pretreatment attenuated curcumin- and cisplatin-induced damage in PTC cells. We observed increased nuclear condensation, distorted membrane integrity, moderate chromatin condensation, and nuclear fragmentation in the cells challenged with both cisplatin and curcumin, while the control and NAC-treated cells depicted diffuse nuclear staining with intact membrane integrity. BCPAP cells were treated with 0 µM or 20 µM of curcumin and 10 µM of cisplatin alone and in combination or NAC (10 mM) and NAC + curcumin and cisplatin for 24 h, and cells were fixed and subsequently stained with 4′,6-diamidino-2-phenylindole (DAPI). Images were taken through an EVOS FLc Cell Imaging System from Invitrogen (Thermo Fisher Scientific) at 40× magnification (scale bar: 100 µm). (**D**) NAC prevented curcumin- and cisplatin-induced modulation in the expression of antiapoptotic and proapoptotic proteins in PTC cells. BCPAP cells were pretreated with NAC (10 mM), followed by a combination treatment of 20 µM curcumin and 10 µM cisplatin, and after cell lysis, equal amounts of proteins were separated by SDS-PAGE, transferred to a PVDF membrane, and immunoblotted with antibodies of Bcl2, Bcl-xL, Bax, and Hsp60. (**E**) NAC prevented curcumin- and cisplatin-induced activation of caspase-mediated apoptosis in PTC cells. BCPAP cells were pretreated with NAC (10 mM), followed by a combination treatment of 20 µM curcumin and 10 µM cisplatin, and after cell lysis, equal amounts of proteins were separated by SDS-PAGE, transferred to a PVDF membrane, and immunoblotted with antibodies of caspase-9, cleaved caspase-8, cleaved caspase-3, PARP, and Hsp60. CUR: curcumin; CIS: cisplatin.

It is well established that oxidative stress due to the overproduction of ROS plays a vital role in cancer therapy; hence, we wanted to explore the involvement of ROS in curcumin- and cisplatin-induced apoptosis in PTC cells. To determine whether ROS are involved in curcumin- and cisplatin-mediated apoptotic cell death in PTC cells, BCPAP and TPC-1 cells were pretreated with 10 mM of NAC, followed by treatment with 0 µM and 20 µM of curcumin and 10 µM of cisplatin alone and in combination for 24 h. Our data revealed that there was a significant increase in the percentage of apoptotic BCPAP cells treated with curcumin and cisplatin compared to individual drug treatment, while NAC pretreatment inhibited apoptosis (Figure 7A, Supplementary Materials, Figure S5E). 

Further, we also detected the level of ROS and morphological alterations using DCFDA and DAPI staining in the BCPAP cells treated with 10 mM NAC, followed by treatment with 0 µM or 20 µM curcumin and 10 µM of cisplatin alone and in combination. Our data revealed that there was a significant increase in the DCFDA staining and in structural alterations in cells treated with both curcumin and cisplatin compared to the individual drug treatment, while NAC pretreatment inhibited DCFDA staining and structural alterations (Figure 7B,C). Similarly NAC treatment attenuated structural alterations due to the curcumin and cisplatin in TPC-1 cells, as shown in the Supplementary Materials, Figure S5F. At a molecular level, we wanted to see the expression of various genes associated with apoptosis, and to do so, BCPAP and TPC-1 cells were treated with 10 mM NAC, followed by treatment with 0 µM and 20 µM of curcumin and 10 µM of cisplatin alone and in combination. Western blot data further supported the involvement of ROS in curcumin- and cisplatin-induced cellular and molecular alterations related to apoptosis in PTC cells, as the change or modulation in the expression of the proapoptotic protein Bax and the antiapoptotic proteins Bcl2, Bcl-xL, caspase-9, cleaved caspase-8, cleaved caspase-3, PARP, and pH2AX was reversed by NAC treatment (Figure 7D,E; Supplementary Materials, Figure S5G). We found that the treatment of PTC cells with curcumin and cisplatin caused reduced Bcl2 expression, while there was a minimum increase in the expression of Bax, as shown in Figure 7D. We did a densitometric analysis and observed that the Bax/Bcl2 ratio increased in the cells treated with curcumin and cisplatin compared to the untreated control cells, while in the case of NAC- and NAC + curcumin + cisplatin-treated cells, we did not find any marked change in the Bax/Bcl2 ratio compared to the control group (Supplementary Materials, Figure S5D).

### 2.7. IL6 Was Important in the Curcumin- and Cisplatin-Induced Downregulation of p-STAT3

**Figure 8:**
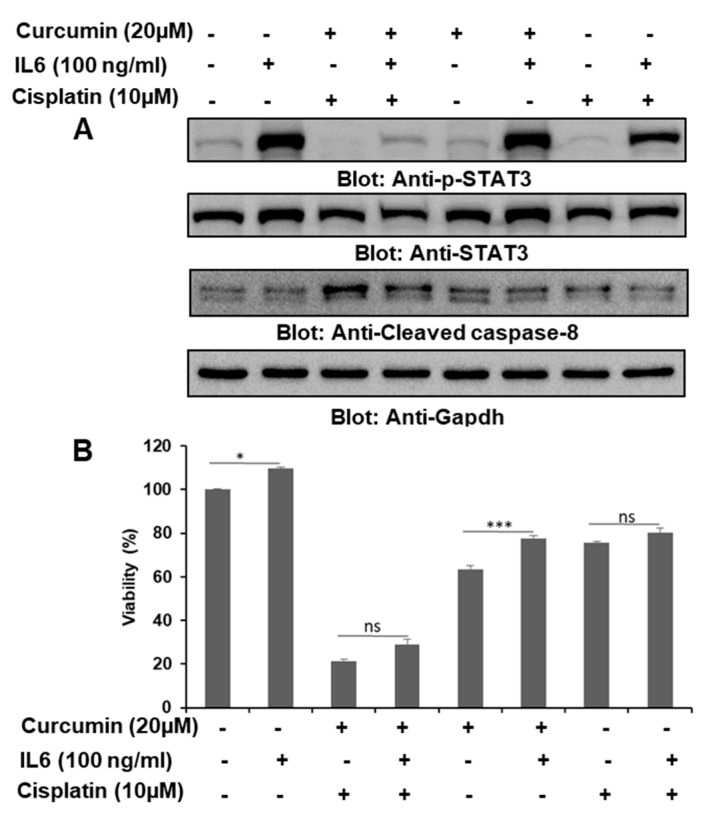
IL6 attenuated the curcumin- and cisplatin-induced inhibition of STAT3 and enhanced the proliferation of PTC cells. (**A**) BCPAP cells were treated with 0 µM or 20 µM of curcumin and 10 µM of cisplatin alone and in combination in reduced serum-containing media, followed by IL6 (100 ng/mL) treatment. Cells were lysed and separated using SDS-PAGE, transferred to a PVDF membrane, and immunoblotted with antibodies such as p-STAT3, STAT3, cleaved caspase-8, and Gapdh. (**B**) IL6 stimulated the cell viability of BCPAP cells and protected cells from curcumin- and cisplatin-mediated inhibition of cell proliferation. BCPAP cells were stimulated with IL6 (100 ng/mL) and subsequently treated with curcumin and cisplatin, as indicated in Figure 2B, for 24 h. A cell proliferation assay was performed using a CCK8 kit, as described in the materials and methods section. Values are expressed as the mean +/− the SD of at least six replicates, with a *p*-value of * *p* < 0.05, *** *p* < 0.001.

IL6 has been shown to mediate its diverse biological functions, such as normal cellular growth and an immune response, through the activation of STAT3, and the dysregulated secretion of IL6 is known to play a crucial role in the pathogenesis of various diseases, including cancer. To assess the role of IL6 in the activation of STAT3 and other regulatory molecules, we treated BCPAP cells with curcumin or cisplatin alone and in combination with and without IL6 (100 ng/mL). We observed that IL6 treatment caused the phosphorylation of STAT3 and a modulation in the expression of CC8, which was associated with cell proliferation and apoptosis (Figure 8A). Overall, our data suggest that IL6 plays an important role in the growth and proliferation of PTC cells, as the exogenous addition of IL6 protected BCPAP cells challenged with 0 µM or 20 µM of curcumin and 10 µM of cisplatin alone and in combination. Further, we also evaluated the role of IL6 (100 ng/mL) in the proliferation of PTC cells treated with 0 µM or 20 µM of curcumin and 10 µM of cisplatin alone and in combination, and our data showed that IL6 significantly enhanced the proliferation of PTC cells compared to control cells (*p* < 0.05) and curcumin-treated cells (*p* < 0.001), while the change in proliferation in cells treated with cisplatin or cisplatin plus IL6 was not significant (Figure 8B).

### 2.8. Cotreatment of Curcumin and Cisplatin Alleviated the Stemness of Thyroid Cancer Stem Cells (CSCs)

**Figure 9:**
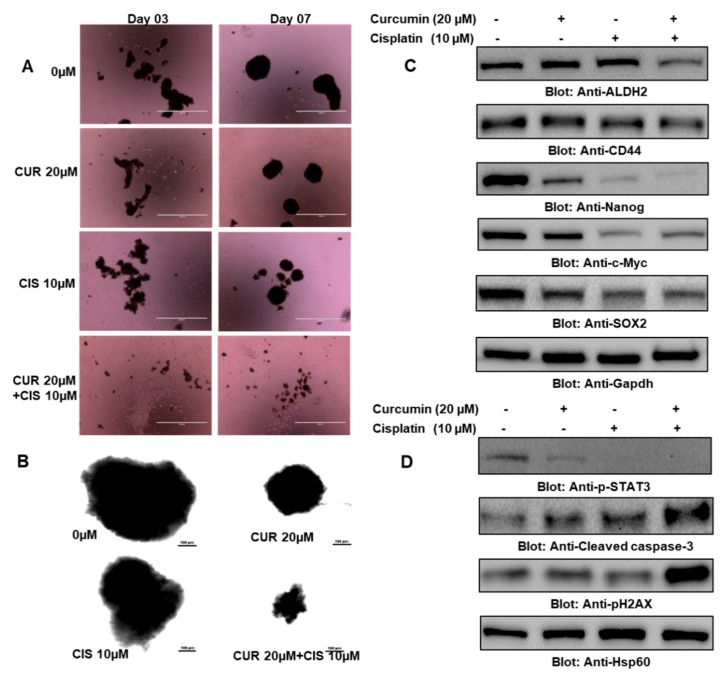
Cotreatment of curcumin and cisplatin potentiated the inhibition of cancer stem cell (CSC) growth and stemness features. (**A**) Treatment with curcumin and cisplatin inhibited thyrosphere formation. BCPAP cells were grown and treated in ultralow attachment plates with 0 µM or 20 µM of curcumin and 10 µM of cisplatin alone and in combination for 7 days, and images of thyrospheres were taken on day 3 and day 7 using an EVOS FLc Cell Imaging System from Invitrogen (Thermo Fisher Scientific) at a magnification of 4×(scale bar 1000 µm). (**B**) BCPAP cells were grown and treated in ultralow attachment plates with 0 µM and 20 µM of curcumin and 10 µM of cisplatin alone and in combination for 7 days, and images of thyrospheres were taken on day 7 through a confocal microscope at a magnification of 20×(scale bar 100 µm). (**C**) Cotreatment of curcumin and cisplatin potentiated the inhibition of stemness features in PTC cells. Thyrospheres were grown and treated in ultralow attachment plates with 0 µM or 20 µM of curcumin and 10 µM of cisplatin alone and in combination, followed by cell lysis and western blotting against ALDH2, CD44, Nanog, c-Myc, SOX-2, and Gapdh. (**D**) Cotreatment with curcumin and cisplatin potentiated the apoptosis of CSCs. Thyrospheres were grown and treated in ultralow attachment plates with 0 µM and 20 µM of curcumin and 10 µM of cisplatin alone and in combination for 7 days, followed by cell lysis and western blotting against p-STAT3, cleaved caspase-3, p-H2AX, and Hsp60. CUR: curcumin; CIS: cisplatin.

CSCs are a small population of cells present in tumors that play a critical role in cancer resistance, relapse, tumorigenesis, and poor clinical outcomes, which compelled us to investigate the effect of 0 µM and 20 µM of curcumin and 10 µM of cisplatin alone and in combination on the growth and stemness of thyroid cancer stem cells. In the first experiment, we plated an equal number of cells collected from thyrospheres that were treated with 0 µM and 20 µM of curcumin and 10 µM of cisplatin alone and in combination, and the growth and size of thyrospheres were evaluated. We found that cells treated with both curcumin and cisplatin had smaller and fewer numbers of thyrospheres compared to the cells treated with either drug alone (Figure 9A,B). Further, our data also revealed that curcumin and cisplatin also suppressed markers of stemness, such as CD44, ALDH, c-Myc, SOX-2, and Nanog (Figure 9C). We also observed that the treatment of thyrospheres with a combination of curcumin and cisplatin was more effective in the induction of apoptosis compared to treatment with either drug alone (Figure 9D). Overall, these findings suggest that curcumin and cisplatin treatment in thyroid cancer cells not only inhibits the growth and proliferation of cancer cells but also reduces the stemness potential of CSCs.

## 3. Discussion

Malignancies of the thyroid gland, e.g., thyroid carcinoma (TC), represents the most prevalent cancer in human endocrine glands [1], although PTC is well differentiated and has a comparatively better prognosis and clinical outcome. Still, there are various concerns related to PTC that need further investigation, such as demonstrating the underlying mechanisms for the aggressiveness of some types of PTC [33,34]. In the current study, we chose curcumin and cisplatin and treated BCPAP and TPC-1 cell lines alone and in combination to decipher the underlying mechanism for the inhibition of the growth and proliferation of PTC cells. Previously, the anticancer potential of curcumin was investigated against thyroid malignancies [35,36] by targeting endoplasmic stress and TGF-β/SMAD; here, for the first time and to the best of our knowledge, we demonstrated the antitumorigenic potential of curcumin alone and in combination with the chemotherapeutic drug cisplatin, as the development of novel therapeutic strategies is imperative for aggressive and refractory PTC, by targeting STAT3. The findings of our study showed that treatment with curcumin alone and in combination with cisplatin markedly inhibited cell proliferation, cell cycle arrest, and apoptosis (Figure 1, Figure 2, Figure 3, Figure 4, Figure 5, Figure 6 and Figure 7). Further, we also demonstrated that the IL6-mediated phosphorylation of STAT3 could be one of the driving forces associated with the proliferation of PTC cells, as it was abrogated by curcumin and cisplatin treatment (Figure 8). Interestingly, we further showed that curcumin and cisplatin treatment also alleviated the stemness of PTC cells (Figure 9). Uncontrolled growth and cell proliferation are crucial cancer hallmarks and directly or indirectly affect the functioning of other regulatory proteins; hence, the targeting of regulatory proteins associated with uncontrolled cell proliferation and survival could be a viable approach for cancer treatment. The JAK/STAT3 signal transduction pathway functions mainly through cytokines and is vital for normal cellular homeostasis, as it regulates cell proliferation, growth, apoptosis, differentiation, and other important cellular biological phenomenons. In most human malignancies, the deregulated or constitutive activation of the JAK-mediated persistent activation of STAT transcription factors has been reported [5,37]. Our findings indicate the antitumorigenic potential of curcumin against PTC cells, as it inhibited cell proliferation and growth by modulating the expression of apoptotic signaling proteins. Furthermore, our data also evidently support the oncogenic potential of deregulated JAK/STAT3 signaling, as we found an overexpression of proteins associated with the inhibition of apoptosis such as Bcl2, Cyclin D1, and Bcl-xL. Furthermore, we also found that the curcumin-induced downregulation of JAK/STAT3 was crucial for the inhibition of growth and the proliferation of PTC cells, which further supports the oncogenic role of the aberrantly activated JAK/STAT3. Our findings also show that curcumin treatment in PTC cells downregulated the expression of CXC chemokine receptor-4 (CXCR4), an important receptor known to play an important role in cell proliferation, migration, and metastasis in thyroid malignancies [38,39].

Further, in the present study, we investigated the combinational effect of curcumin and cisplatin on the survival and proliferation of PTC cells in vitro by targeting JAK/STAT3 signaling. A number of reports have revealed that the cytokine-induced activation of STAT3 is associated with the evasion of apoptosis, among other cancer hallmarks [6,7,8]. There is a strong correlation between STAT3 overexpression and the pathogenesis of thyroid cancer, as the dysregulated activation of STAT3 has been reported in human specimens of thyroid cancers [9,10]. Apoptosis, or programmed cell death is an important phenomenon that maintains normal cell proliferation and growth (regulated by a system of interconnected signals that is often dysregulated in most human pathologies, including cancer) [40]. Caspases are the main executers of apoptosis and are activated by upstream signaling (such as STAT3), as has been reported for different human malignancies such as breast, blood, and thyroid cancers [41,42,43]. The induction of apoptosis by curcumin and cisplatin in various cancer types induces caspase activation and cleavage via different mechanisms, including ROS generation [44,45]. In the present study, we found that the treatment of PTC cells with curcumin and cisplatin caused morphological alterations, cell cycle arrest, a reduction in the mitochondrial membrane potential and DNA double-strand breaking, the activation of caspases, and an increased Bax/Bcl2 ratio, while NAC treatment reversed all of these changes, suggesting the critical role of ROS. All of these cellular and molecular changes support the potential role of ROS in the curcumin- and cisplatin-mediated inhibition of the growth and proliferation of PTC cells. Bax is an antiapoptotic gene that is often under expressed in human malignancies, and it is well known for its role in cancer cell survival and resistance [46]: it has been shown that increased Bax expression and the reduced expression of Bcl2 are important underlying mechanisms of apoptosis [47,48]. We found that curcumin and cisplatin treatment in PTC cells inhibited Bcl2 expression and elevated Bax expression (in other words, increasing the Bax/Bcl2 ratio), which supports earlier findings underlying apoptosis in cancer cells [47,48]. The activation of caspases (caspase-3, caspase-8, and caspase-9) and PARP and the downregulation of IAPs provide the molecular basis for the curcumin- and cisplatin-induced cellular death of PTC cells. Aberrantly expressed STAT3 is known to elevate the transcriptional gene expression of proteins implicated in various hallmarks of cancer development, including uncontrolled cell growth and survival [49,50]. Compelling evidence suggests that constitutive STAT3 expression promotes cell survival by modulating the expression of genes associated with cell cycle regulation and growth, such as those involved in the downregulation of CDKIs, those involved in the upregulation of cyclins, antiapoptotic molecules Bcl-xL/Bcl-2, and members of the inhibitors of the apoptosis family (e.g., Survivin and cIAPs) [49,51,52]. The present investigation’s findings reveal that abrogation of the deregulated expression of STAT3 after curcumin and cisplatin treatment inhibited the growth and proliferation of PTC cancer cells via the downregulation of proteins associated with the cell cycle arrest and apoptosis, which further supports earlier demonstrations. Furthermore, the genetic knockdown of STAT3 in PTC cells alleviated the expression of proteins related to uncontrolled cell proliferation and growth.

Since the role of cytokines, particularly IL6, has been well elucidated in terms of STAT3-mediated oncogenesis in various human cancer types, including thyroid cancer, and since it is considered to be a plausible target in the development of cancer therapeutics [30,53,54], we also investigated the role of IL6/STAT3 signaling in PTC cells treated with curcumin and cisplatin. We found that IL6 markedly stimulated STAT3, which in turn activated signaling associated with cell proliferation, as the treatment of PTC cells with curcumin and cisplatin was able to abolish the proliferative action of IL6 compared to treatment with curcumin and cisplatin alone, suggesting that IL6 could be critical in the curcumin- and cisplatin-induced downregulation of STAT3. Hence, this provides further evidence for IL6 and STAT3 being viable targets for cancer therapy. Natural products have been shown to attenuate or suppress various diseases, including cancer, through immunomodulation [55] (and in line with that, through immunotherapy by targeting immune cells, which are the key cells involved in the immune escape of tumor cells, and curcumin has been shown to enhance therapeutic outcomes by targeting regulatory T cells [56,57]. 

Cancer stem cells are a small population of tumor cells that are integral to the pathogenesis of neoplasm and to drawbacks such as drug resistance, cancer relapse, or the recurrence of therapeutic clinical options; hence, targeting these cells is imperative for effective therapeutic outcomes. Therefore, we further checked the effect of curcumin and cisplatin against PTC by targeting the stemness features of thyroid cancer stem cells. 

Our data suggest that curcumin and cisplatin treatment alleviated stemness and the growth and proliferation of thyroid cancer stem cells, which further supports their cancer therapeutic potential, most likely through the attenuation of HIF-1-alpha as earlier reports showed that curcumin inhibits the growth and proliferation of cancer stem cells and drug resistance via the degradation of HIF-1-alpha [58,59,60,61].

## 4. Materials and Methods

### 4.1. Reagents and Antibodies

Curcumin, cisplatin, IL6, and all other chemicals were purchased from Sigma Aldrich (St. Louis, MO, USA). Phospho-STAT3, STAT3, phospho-JAK2, JAK2, phospho-H2AX, phospho-CXCR4, Caspase-9, Cleaved caspase-9, Cleaved caspase-8, Caspase-3, Cleaved caspase-3, PARP, XIAP, cIAP1, cIAP2, Bcl-xL, Bax, p27, p21, Survivin, c-Myc, Nanog, Aldh, Gapdh, etc., were purchased from Cell Signaling Technologies (3 Trask Lane, Danvers, MA, USA). SOX2, Hsp60, and Bcl2 antibodies were procured from Santa Cruz Biotechnology (Finnell Street Dallas, TX, USA). Annexin V-FITC, propidium iodide staining solution, Hoechst 33342 Solution, BD Cytofix/Cytoperm plus fixation, and apermeabilization solution kit (a BD MitoScreen (JC-1) Kit) were purchased from BD Biosciences (Qume Drive, San Jose, CA, USA).

### 4.2. Cell Lines and Cell Culture 

The human papillary thyroid carcinoma cell (PTC) cancer cell lines, BCPAP and TPC-I, were obtained from DSMZ (Braunschh, Germany) and EMD Millipore, MA, USA, respectively, and were cultured using RPMI 1640 medium supplemented with 10% (*v*/*v*) fetal bovine serum (FBS), 100 U/mL penicillin, and 100 U/mL streptomycin at 37 °C in a humidified atmosphere containing 5% CO2. Glutamax (1%) added to TPC-1 culture media and 5% RPMI media was used in all of the experimental treatments. 

### 4.3. Measurement of Real-Time Cell Proliferation (Cell Index) of PTC Cells Treated with Curcumin, Cisplatin, and Curcumin + Cisplatin Using an RTCA xCELLigencecell Analyzer

Briefly, BCPAP and TPC-1 cells were grown for 24 h in a monolayer on top of electrodes and then were treated with the indicated concentrations of curcumin, cisplatin alone, and in combination. A real-time cell analyzer and E-plate 16 (RTCA; xCELLigence, Roche, San Diego, CA, USA) were used to determine the cell viability of curcumin-, cisplatin-, and curcumin + cisplatin-treated and -untreated cultured cells using electrical impedance [62]. 

### 4.4. Cell-Counting Kit-8 (CCK8) Assay 

The growth inhibitory and antiproliferative potential of curcumin and cisplatin alone and in combination on the PTC cell lines BCPAP and TPC-1 were investigated by using a cell-counting kit-8 reagent, as described previously [63]. In short, 10^4^ cells were incubated in a 96-well plate for 24 h and then treated with varying doses of curcumin and cisplatin alone and in combination for 24 h. After that, cell-counting kit-8 solution was added per the manufacturer’s instructions, followed by incubation at 37 °C. Finally, the optical density (OD) was recorded at 450 nm. Percent cell viability was calculated as the OD of the experiment samples/the OD of the control sample × 100.

### 4.5. Cell Migration and Colonogenic Assay

A cell migration and colonogenic assay was performed as described previously [64]. Briefly, cells were at ~80% confluence, and a fine scratch in the form of a groove was created with a sterile fine tip, followed by imaging (denoted as 0 h). Further, cells were treated with curcumin and cisplatin alone and in combination and were allowed to grow for 24 h. Thereafter, imaging was done, and the time point was designated as 24 h. For colony formation, BCPAP cells, around 10^4^, were plated in a six-well plate, followed by treatment with curcumin and cisplatin alone and in combination. Next, the plates were monitored for one to two weeks, and finally, the colonies were stained and examined microscopically for further analysis using an EVOS FLc Cell Imaging System from Invitrogen (Thermo Fisher Scientific, Waltham, MA, USA). 

### 4.6. Annexin V Staining

PTC cells (BCPAP) were treated with the indicated concentrations of curcumin and cisplatin alone and in combination, followed by incubation for 24 h. Cells were harvested, washed with PBS, and then stained with flourescein-conjugated Annexin V and propidium iodide. Finally, apoptosis was measured as described previously [65,66] by flow cytometry (BD LSRFortessa Analyzer, BD Biosciences, Qume Drive, San Jose, CA, USA). 

### 4.7. Cell Lysis and Immunoblotting

Following the treatment with varying doses of curcumin and cisplatin alone and in combination, cells were harvested and lysed, as described previously [67]. An equal amount of proteins was separated using SDS-PAGE and transferred to a polyvinylidene difluoride (PVDF) membrane. Specific antibodies were used against proteins of interest and were immunoblotted and further visualized under a ChemiDoc System (Bio-Rad, Hercules, CA, USA).

### 4.8. Measurement of Mitochondrial Membrane Potential

Appropriate numbers of cells were treated with curcumin and cisplatin alone and in combination for 24 h. After the treatment, cells were harvested, washed, and finally stained with a JC1 stain kit per the manufacturer’s instructions: they were then analyzed using flow cytometry (BD LSR Fortessa Analyzer, BD Biosciences, Qume Drive, San Jose, CA, USA).

### 4.9. Gene Silencing Using siRNA

STAT3 siRNA (catalogue no.: 4390824; lot no.: AS02B109, Ambion, Thermo Fisher Scientific, Waltham, MA, USA) and scrambled control siRNA (catalogue no.: 1027281) were obtained from Qiagen. BCPAP cells were transfected using Lipofectamine 2000 reagent (Invitrogen, Thermo Fisher Scientific, Waltham, MA, USA) according to the manufacturer’s instructions. The lipid and siRNA complex was removed after 6 h, and cells were supplemented with complete medium and incubated for 48 h. Cells were lysed and immunoblotted with various antibodies.

### 4.10. Thyrosphere-Forming Assay and Stemness Profile

Thyrospheres containing cancer stem cells were generated in the form of spheres, as described earlier [68]. Briefly, BCPAP cells were cultured in 5% DMEM media first, followed by enzymatic treatment, to make a single cell suspension. Then, a specific number of cells were cultured in DMEM-F12 medium supplemented with b27 (Life Technologies, Thermo Fisher Scientific, Waltham, MA, USA) and growth factors such as epidermal growth factor (EGF) and basic fibroblast growth factor (bFGF) (Sigma Aldrich Inc., St. Louis, MO, USA). To evaluate the effect of curcumin and cisplatin on the stemness of PTC cells, we treated the thyrospheres and analyzed the stemness markers. 

### 4.11. Statistical Analysis

Comparisons between groups were made using one-way ANOVA followed by a paired Student’s *t*-test using the software GraphPad Prism (version 5.0 for Windows, GraphPad Software Inc., San Diego, CA, USA, http://www.graphpad.com). Values of * *p* < 0.05 were considered statistically significant. 

## 5. Conclusions

Overall, the findings of the present study evidently support the better anticancer potential of a combination treatment of curcumin and cisplatin in thyroid cancer cells, which suggests that a combination treatment of curcumin and cisplatin may provide better cancer therapeutic outcomes. The attenuation of aberrantly activated JAK/STAT through curcumin and cisplatin treatment was critical to the inhibition of the proliferation and growth of PTC cells. Interestingly, IL6 played a critical role in the dysregulation of STAT3 and may be involved in protection against apoptotic cell death in PTC cells. Additionally, curcumin and cisplatin also inhibited the stemness of thyroid cancer cells. 

## Supplementary Materials

Supplementary materials can be found at https://www.mdpi.com/1422-0067/21/2/438/s1.
