# Supplementary material for: Curcumin-Mediated Apoptotic Cell Death in Papillary Thyroid Cancer and Cancer Stem-Like Cells through Targeting of the JAK/STAT3 Signaling Pathway
Source: Int J Mol Sci. 2020 Jan 9;21(2):438. doi: 10.3390/ijms21020438 (PMC7014270; doi:10.3390/ijms21020438)
Supplement: Supplementary file 1 [file ijms-21-00438-s001.pdf]

Supplementary Materials

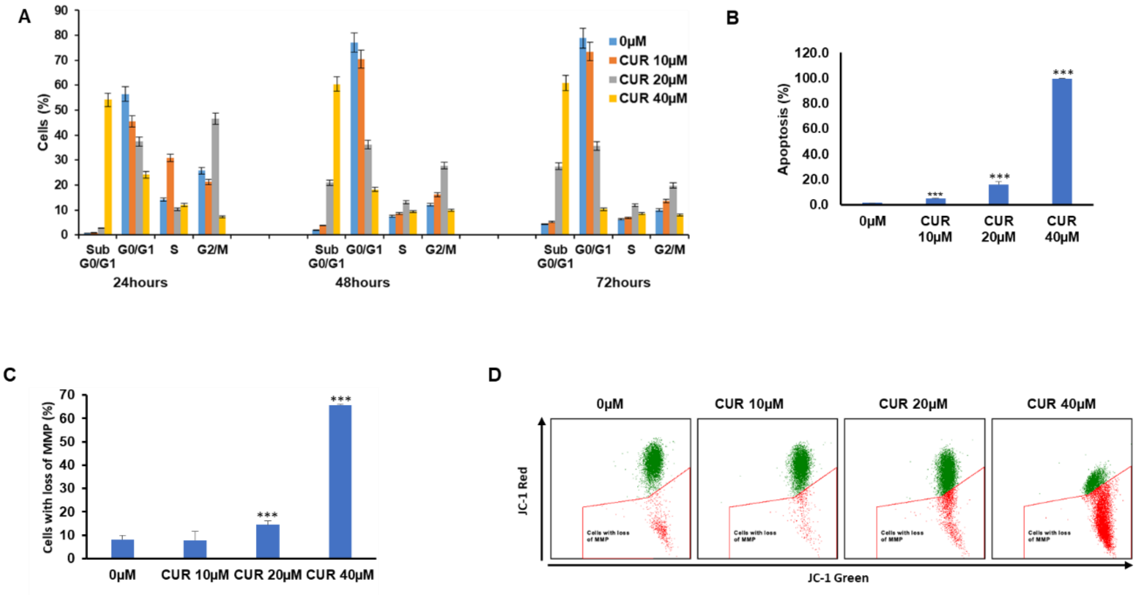

Supplementary Figure S1: **(A).** Curcumin-induced cell cycle arrest in PTC cells. BCPAP cells ware treated with 10µM, 20µM, and 40µM curcumin for 24 hours, 48 hours and 72 hours and cells were processed for the cell cycle analysis by flow cytometry. Curcumin treatment markedly enhanced SubG0 fraction in BCPAP cells in all three time points. **(B**). Curcumin-induced apoptosis in PTC cells. BCPAP cells were treated with 10µM, 20µM, and 40µM for 24 h and cells were subsequently stained with fluorescein-conjugated annexin-V and propidium iodide (PI) and analyzed by flow cytometry and apoptosis was measured by flow cytometry after staining with annexin-V and PI and percentage of apoptosis relative to untreated cells was calculated ($p<0.05$). **(C-D).** Curcumin treatment causes loss of MMP in PTC cells. BCPAP cells were treated with indicated doses of curcumin for 24 h. After JC1 staining, cells were analyzed by flow cytometry as described. Values are expressed as mean ± SD and P value <0.05 was considered as significant. CUR-Curcumin

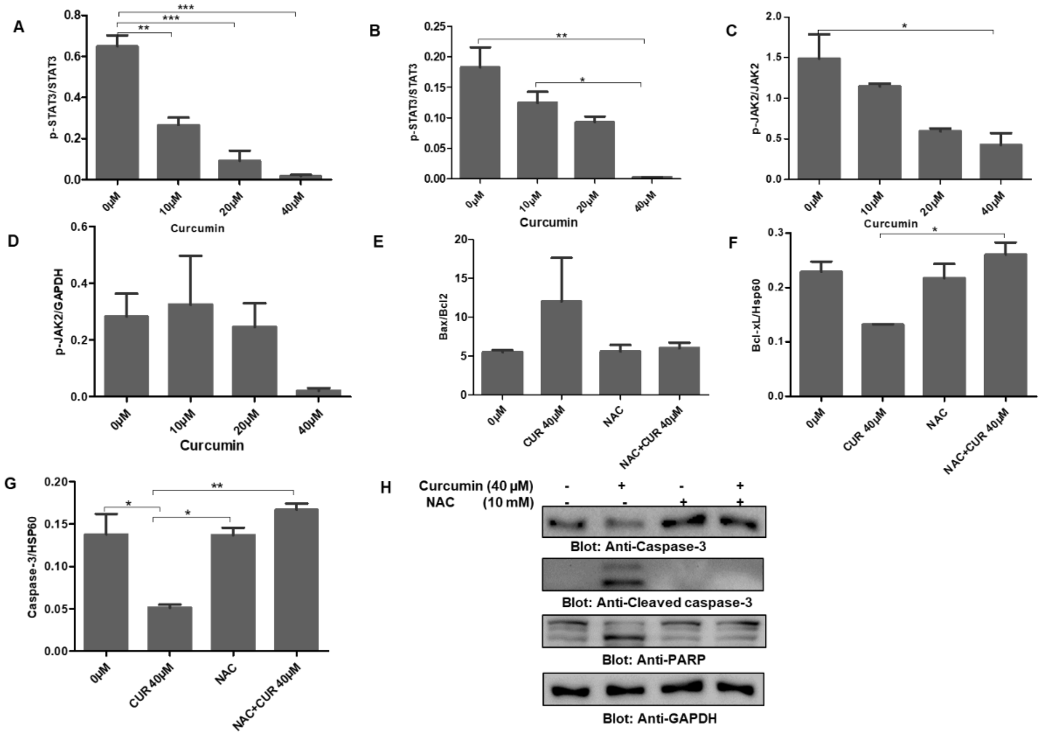

Supplementary Figure S2: (A and B) Densitometric analysis of p-STAT3 in BCPAP and TPC-1 cells treated with increasing doses of curcumin for 24 hours respectively. After cell lysis, equal amounts of proteins were separated by SDS–PAGE, transferred to PVDF membrane and immunoblotted with antibodies against pSTAT3, STAT3. Data expressed as relative density normalized with total STAT3 and represented as mean ± SD (*p<0.05, **p<0.01,***p<0.001). **(C and D)** Densitometric analysis of p-JAK2, normalized with total JAK2 and GAPDH in BCPAP and TPC-1 cells respectively, treated with increasing doses of curcumin for 24 hours respectively. Cells were lysed and equal amounts of proteins were separated by SDS–PAGE, transferred to PVDF membrane and immunoblotted with antibodies against p-JAK2 and JAK2 and GAPDH. Data expressed as relative density normalized with total JAK2 and GAPDH and represented as mean ± SD (*p<0.05). **(E).** Densitometric analysis of Bcl2 and Bax in BCPAP cells treated with curcumin and NAC alone and in combination for 24 hours. Cells were lysed and equal amounts of proteins were separated by SDS–PAGE, transferred to PVDF membrane and immunoblotted with antibodies against Bcl2 and Bax. Data expressed as relative density normalized with GAPDH in the ratio of Bax and Bcl2 and represented as mean ± SD. **(F and G).** Densitometric analysis of Bcl-xL and caspase-3 in BCPAP cells treated with curcumin and NAC alone and in combination for 24 hours, respectively. Cells were lysed and equal amounts of proteins were separated by SDS–PAGE, transferred to PVDF membrane and immunoblotted with antibodies against Bcl-xL, caspase3 and shp60. Data expressed as relative density normalized with SHP60 and represented as mean ± SD (*p<0.05, **p<0.01). **(H).** NAC treatment inhibits curcumin-induced apoptosis in TPC-1 cells. TPC-1 cells were treated with curcumin (40μM) with and without NAC for 24 hrs then cells were lysed and equal amounts of proteins were separated by SDS–PAGE, transferred to PVDF membrane and immunoblotted with antibodies against caspase3, cleaved caspase-3, PARP and GAPDH. CUR-Curcumin; NAC-N-Acetyl Cysteine

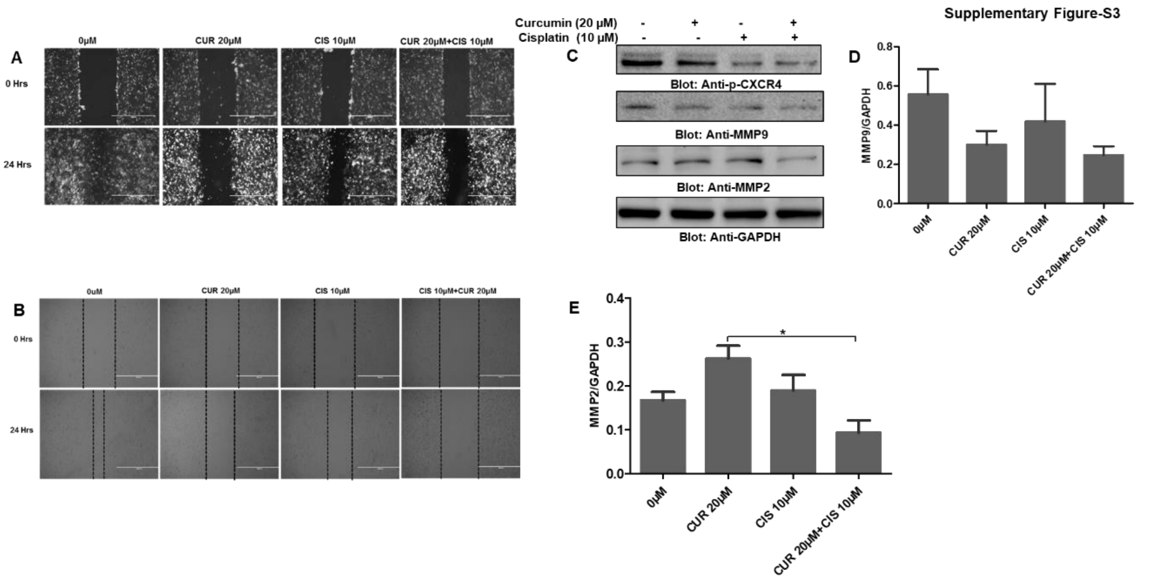

Supplementary Figure S3: Co-treatment of curcumin and cisplatin suppress migration of PTC cell lines BCPAP (**A**) and TPC-1 (**B**). BCPAP and TPC-1 cells were grown in six well plate, grooves were created with sterile tip followed by treatment with 20μM curcumin and 10μM cisplatin alone and in combination for 24 hours. Cell migration or gap filled due to cell migration was examined under EVOS FLc Cell Imaging System from Invitrogen (Thermo Fisher Scientific) (4x-magnification; scale bar 1000 μm) (**C**). Curcumin and cisplatin co-treatment downregulated p-CXCR4, and matrix metalloproteinase. BCPAP cells were treated with 0μM, 20μM curcumin and 10μM cisplatin alone and in combination for 24 hours. Cells were lysed and equal amount of proteins were separated on SDS-PAGE, transferred to PVDF membrane, and immunoblotted with antibodies such as pCXCR4, MMP9, MMP2, and GAPDH. (**D, E**). Densitometric analysis of MMP9 and MMP2. Data expressed as relative density of each protein normalized with GAPDH and represented as mean ± SD (*$p<0.05$). CUR-Curcumin; CIS-Cisplatin

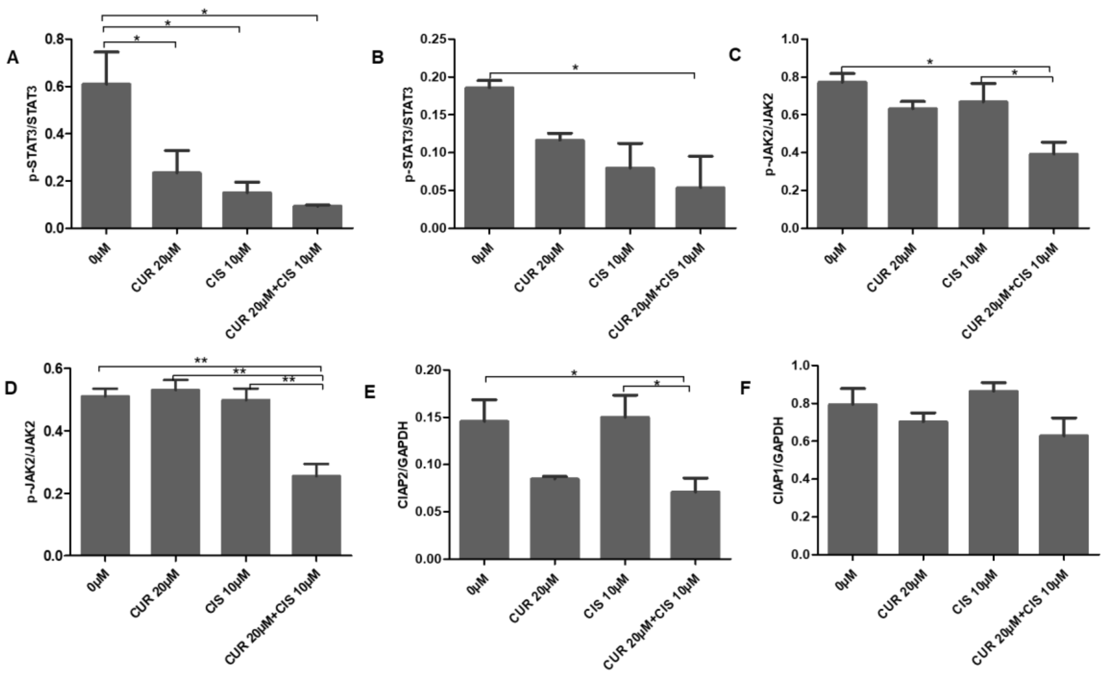

Supplementary Figure S4: Co-treatment of curcumin and cisplatin inhibits JAK/STAT3 pathway. **A** and **B** represents densitometry data of p-STAT3 expression normalized with total STAT3 in BCPAP and TPC-1 cells respectively, treated with 0μM, 20μM curcumin and 10μM cisplatin alone and in combination for 24 hours. Data were expressed as mean ± SD. (*p<0.05). **C** and **D** represents densitometry data of pJAK2 expression normalized with total expressed JAK2 in BCPAP and TPC-1 cells respectively, treated with 0μM, 20μM curcumin and 10μM cisplatin alone and in combination for 24 hours. Data were expressed as mean ± SD. (*p<0.05, **p<0.01). **E** and **F** represents densitometry data of CIAP2 and CIAP1 expression normalized GAPDH in BCPAP cells respectively, treated with 0μM, 20μM curcumin and 10μM cisplatin alone and in combination for 24 hours. Data were expressed as mean ± SD. (*p<0.05). CUR-Curcumin; CIS-Cisplatin

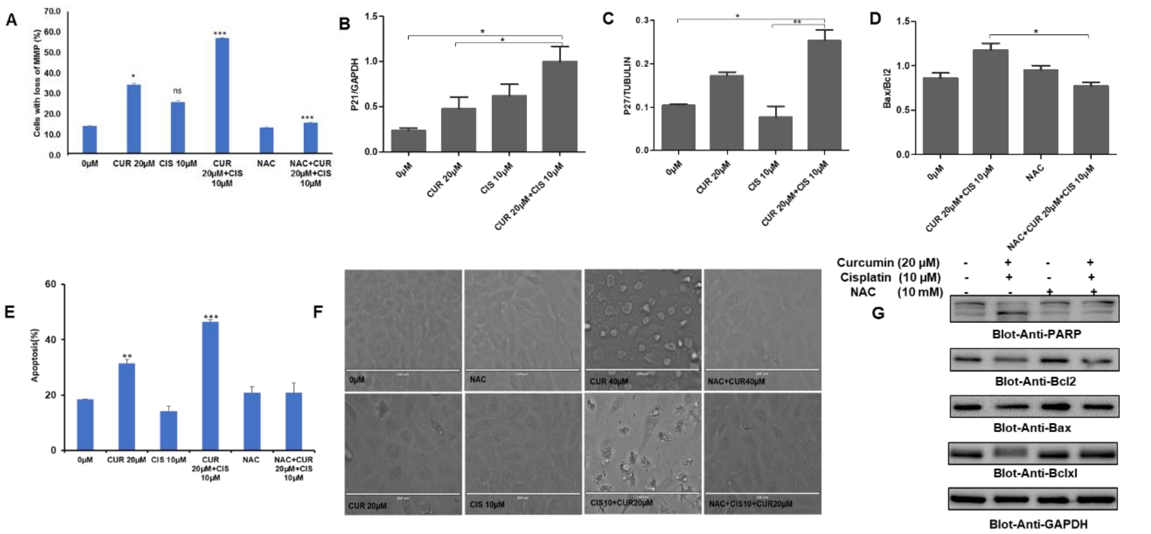

Supplementary Figure S5: **(A)** Co-treatment of curcumin and cisplatin enhanced loss of mitochondrial membrane potential (MMP) and reversed by NAC. BCPAP cells were treated with 0µM, 20µM curcumin, 10mM NAC and 10µM cisplatin alone and in combination for 24 hours and stained with JC1 and then analyzed using flow cytometry. Data were expressed as mean and S.D. (**p<0.01, ***p<0.001). (**B**) and (**C**) represents densitometry data of p21 and p27 expression in BCPAP cells treated with 0µM, 20µM curcumin and 10µM cisplatin alone and in combination for 24 hours normalized with GAPDH and tubulin respectively. Data were expressed as mean and S.D. (*p<0.05, **p<0.01). **(D).** Represents densitometry data of Bax/Bcl2 ratio expression in BCPAP cells treated with 10mM NAC, 20µM curcumin and 10µM cisplatin alone and in combination for 24 hours normalized with GAPDH. Data were expressed as mean and S.D. (*p<0.05). **(E)** Co-treatment of curcumin and cisplatin enhanced apoptotic cell death in PTC cells and reversed by NAC. BCPAP cells were treated with 0µM, 20µM curcumin, and 10mM NAC and 10µM cisplatin alone and in combination for 24 hours and stained with Annexin V and Propidium Iodide and then analyzed by flow cytometry. Data were expressed as mean and S.D. (**p<0.01, ***p<0.001). **(F).** NAC treatment reversed cellular alterations in PTC cells. TPC-1 cells were treated with 0µM, 20µM curcumin, 40µM curcumin, 10mM NAC and 10µM cisplatin alone and in combination for 24 hours. Photomicrographs (EVOS FLc Cell Imaging System from Invitrogen (Thermo Fisher Scientific), 20x-maginification; scale bar: 200um) analysis showed that NCA treatment inhibits cellular alterations due to curcumin and cisplatin. **(G).** NAC treatment prevents apoptosis due to curcumin and cisplatin alterations in PTC cells. TPC-1 cells were treated with 0µM, 20µM curcumin, 10mM NAC and 10µM cisplatin alone and in combination for 24 hours. After cell lysis, equal amounts of proteins were separated by SDS–PAGE, transferred to PVDF membrane and immunoblotted with antibodies against PARP, Bcl2, Bax, Bcl-xL and GAPDH. CUR-Curcumin; CIS-Cisplatin; NAC-N-Acetyl Cysteine
